# Genome-Wide H3K4me3 Analysis in Angus Cattle with Divergent Tenderness

**DOI:** 10.1371/journal.pone.0115358

**Published:** 2015-06-18

**Authors:** Chunping Zhao, José A. Carrillo, Fei Tian, Linsen Zan, Scott M. Updike, Keji Zhao, Fei Zhan, Jiuzhou Song

**Affiliations:** 1 College of Animal Science and Technology, Northwest A&F University, Yangling, Shaanxi, China; 2 Department of Animal & Avian Sciences, University of Maryland, College Park, MD, United States of America; 3 Laboratory of Molecular Immunology, National Heart, Lung and Blood Institute, National Institutes of Health, Bethesda, MD, United States of America; University of Bonn, Institut of experimental hematology and transfusion medicine, GERMANY

## Abstract

Tenderness is one of the most important properties of meat quality, which is influenced by genetic and environmental factors. As an intensively studied epigenetic marker, histone methylation, occurring on arginine and lysine residues, has pivotal regulatory functions on gene expression. To examine whether histone methylation involves in beef tenderness variation, we analyzed the transcriptome and H3K4me3 enrichment profiles of muscle strips obtained from the *longissimus dorsi* (LD) of Angus steers previously classify to the tender or tough group. We first plotted a global bovine H3K4me3 map on chromosomes and called peak-enriched regions and genes. We found that majorities of H3K4me3 on genes were occupying the first intron and intergenic regions and its maps displayed similar patterns in tender and tough groups, with high H3K4me3 enrichment surrounding the transcription start site (TSS). We also explored the relationship of H3K4me3 and gene expression. The results showed that H3K4me3 enrichment is highly positively correlated with gene expression across the whole genome. Cluster analysis results confirmed the relationship of H3K4me3 enrichment and gene expression. By using a pathway-based approach in genes with H3K4me3 enrichment in promoter regions from the tender cluster, we revealed that those genes involved in the development of different tissues–connective tissue, skeletal and muscular system and functional tissues–; while in tough group those genes engaged in cell death, lipid metabolism and small molecule biochemistry. The results from this study provide a deep insight into understanding of the mechanisms of epigenetic regulations in meat quality and beef tenderness.

## Introduction

Tenderness is one of the most important factors influencing beef palatability, and is determined cooperatively by genetic and environmental factors [1–3]. Several studies, and especially the ones that examined histone modifications, reported that environmental factors might impact muscle development and beef tenderness through distinct types of epigenetic mechanisms. [4–6]. Histone methylation occurring on arginine and lysine residues has pivotal regulatory functions and has been examined intensively. So far, a lot of research focused on the association of histone methylation and the process of myogenesis, muscular proliferation, differentiation and fusion to form new muscle fibers has been performed. Some reports mentioned that histone H3 lysine 27 tri-methylation (H3K27me3) distributes widely throughout the genome in both myoblasts and myotubes, and could regulate myogenic differentiation through silencing muscle-specific and cell cycle genes [7,8]. Other studies have shown that H3K9me3 participates in shutting off cell cycle genes during muscle differentiation [9]. Researchers also found that the *Myog* (Myogenin) gene displayed enrichment in both H3K4me2 in the promoter region and H3K4me3 within the transcribed region, during active myogenesis [10]. Histone methylation also relates to the muscle fiber type [11]. Differential histone modification patterns were observed at myosin heavy chain genes in fast and slow skeletal muscle fibers [12]. These histone modification studies mainly focused on the key genes, which have been known to play pivotal roles on muscle proliferation and differentiation.

In recent years, genome-wide epigenetic maps have been reported for yeast, mice, fly, bovine and human [13,14]. However, few whole-genome histone modification maps have been created for bovine [13]. In our study, we used chromatin immunoprecipitation along with massively parallel sequencing (ChIP-Seq) to generate particular genome-wide H3K4me3 profiles in Angus cattle with divergent tenderness of *longissimus dorsi* (LD). We correlated gene expression profile with H3K4me3 pattern in promoter regions. Taken together, we intended to find some underlying contribution of histone modification to beef quality. Our results show that H3K4me3 correlates positively with gene expression and particularly enrichment in promoters associates with active genes. We also revealed the relationship of some H3K4me3 markers with the beef tenderness variation.

## Results

### Genome-wide H3K4me3 maps in LD of Angus Cattle

To detect the distribution of histone modification across the entire genome, first we produced a H3K4me3 map employing the ChIP-Seq approach in the tender and tough groups, respectively. After immunoprecipitation, the following steps have been performed successively; ChIP-DNA quality assesment, library construction and sequencing on the Solexa platform. The resulting reads were aligned to a reference genome (bosTau4) and duplicates were removed from the dataset. A total of 3,141,135 unique reads of 25 bp generated from the two representative groups, mapped back to the bovine genome. To further explore the distribution pattern of H3K4me3 on LD muscle, SICER, a commonly used clustering approach for identification of enriched domains from histone modification ChIP-Seq data, was used [8]. In total, we found 12,452 and 11,395 H3K4me3 peaks in tender and tough groups, respectively. From these, 2,710 and 1,829 corresponded for unique peaks in the tender and tough groups. The common peaks accounted 9,742 and 9,566, respectively (Table 1). The different length of peaks in each group could explain the variation in the number of shared peaks; many times a large peak appeared in one group while multiple small peaks, possibly belonging to the same enrichment, occurred in the other group at the same genomic location. The ChIP quality and the peaks were validated through quantitative PCR.

**Table 1:** Genome-wide H3K4me3 distribution in LD of Angus cattle.

Group	Total peaks	Shared peaks	Unique peaks	Genes	Unique genes	Shared genes
Tender	12452	9742	2710	1597	1585	12
Tough	11395	9566	1829	574	562	12

**Fig 1:**
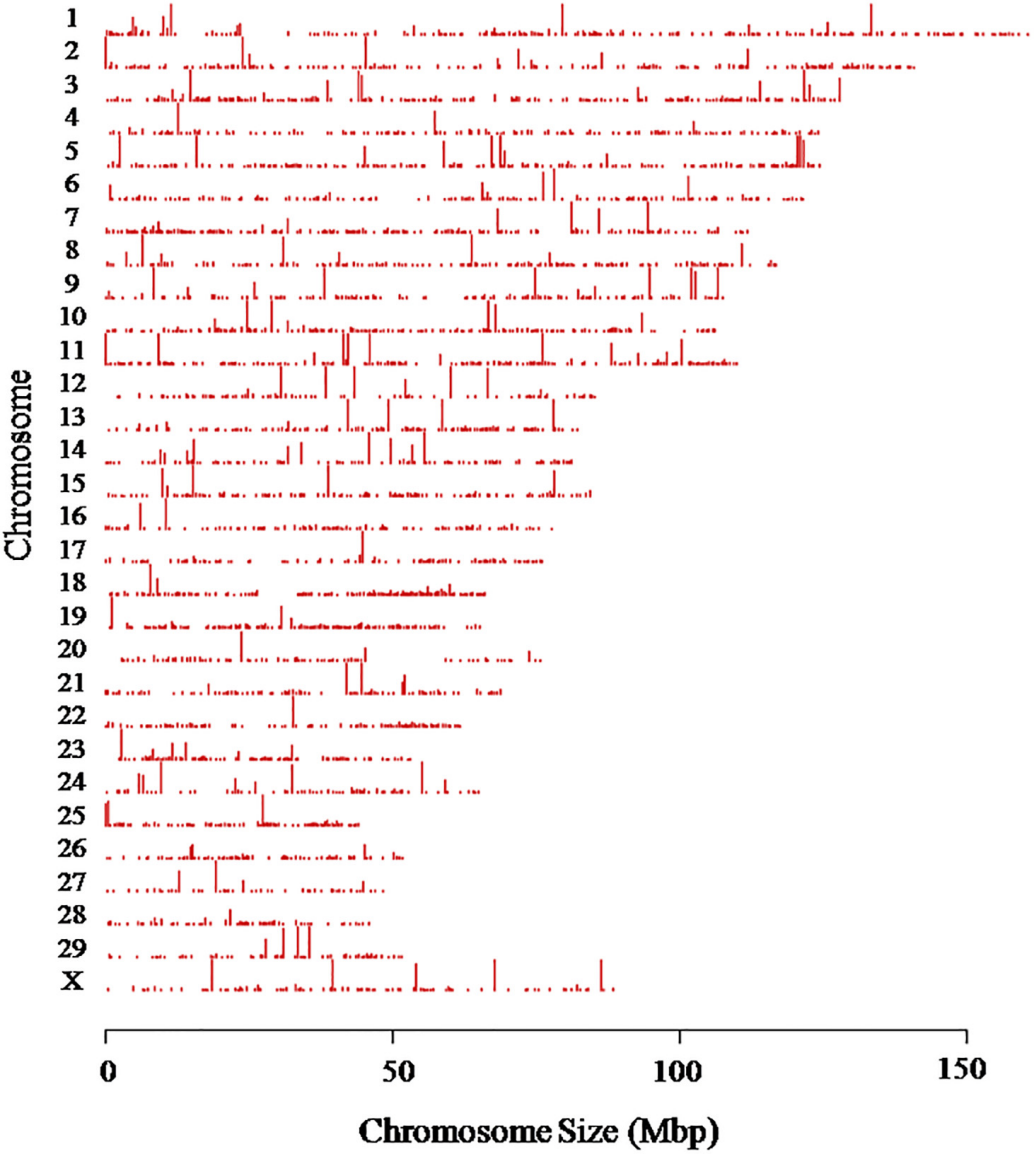
Global distribution of H3K4me3 on the whole genome in LD.

**Fig 2:**
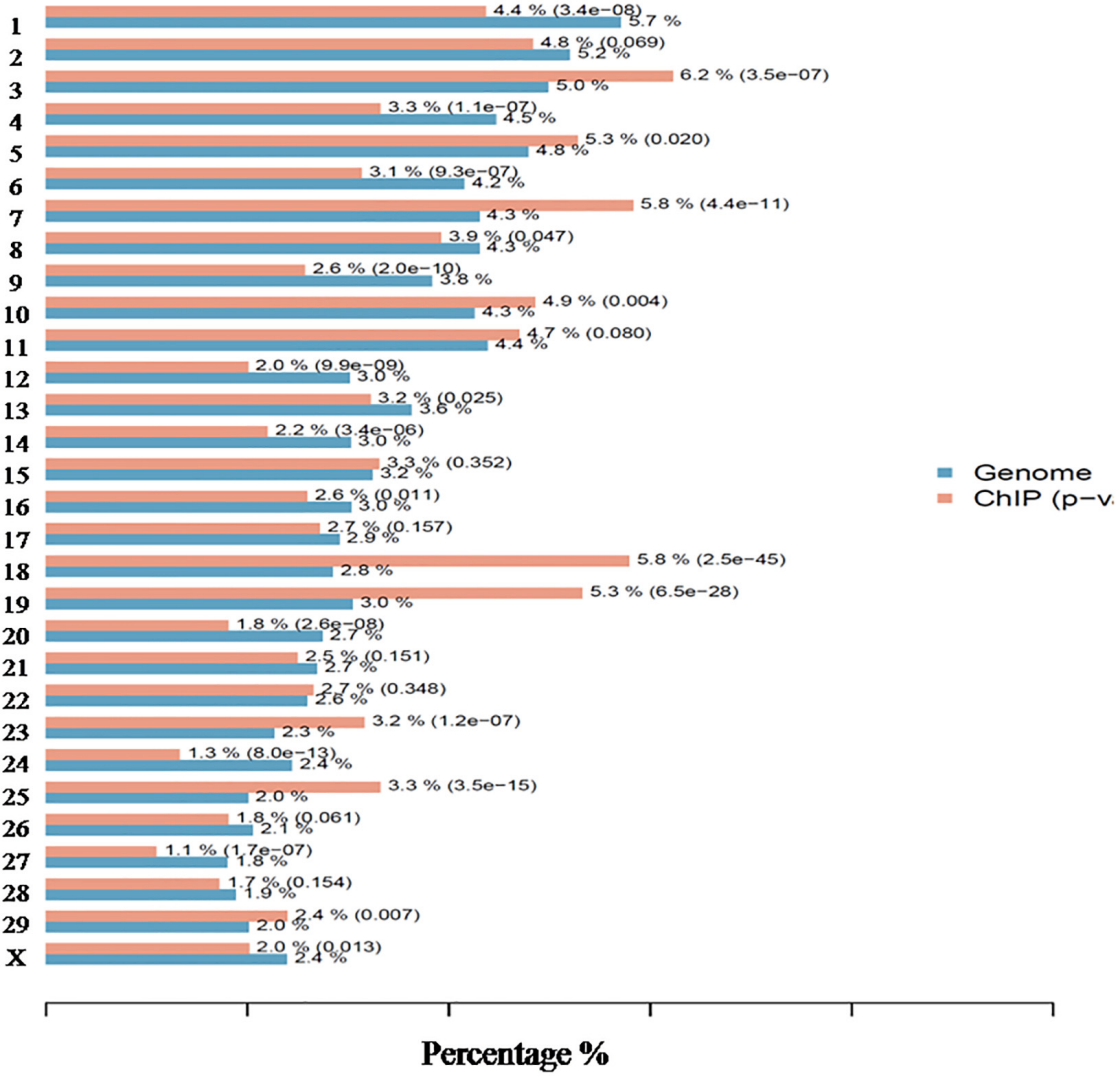
Relative distribution of H3K4me3 on the whole genome in LD.

To obtain the distribution of unique reads for H3K4me3 enrichment on each chromosome, we combined all the unique reads from both tender and tough groups, and then aligned the enriched regions to the bovine genome. The peak distribution on each chromosome was plotted in the Fig 1 and percentages of enrichment compared with the relative lengths of each chromosome are showed in the Fig 2. From the peak distribution, we found that H3K4me3 marker occur heterogeneously on the whole genome. And we further realized that more enrichment of H3K4me3 existed on chromosome 3, 7, 18, 19 and 25 than other chromosomes. These distribution maps implied that genes from these chromosomes are more closely associated with this positive histone marker and may have higher expression levels compared to genes located in other chromosomes. This could also indicate a higher density of LD-specific genes in these chromosomes that are active or could potentially be activated.

**Fig 3:**
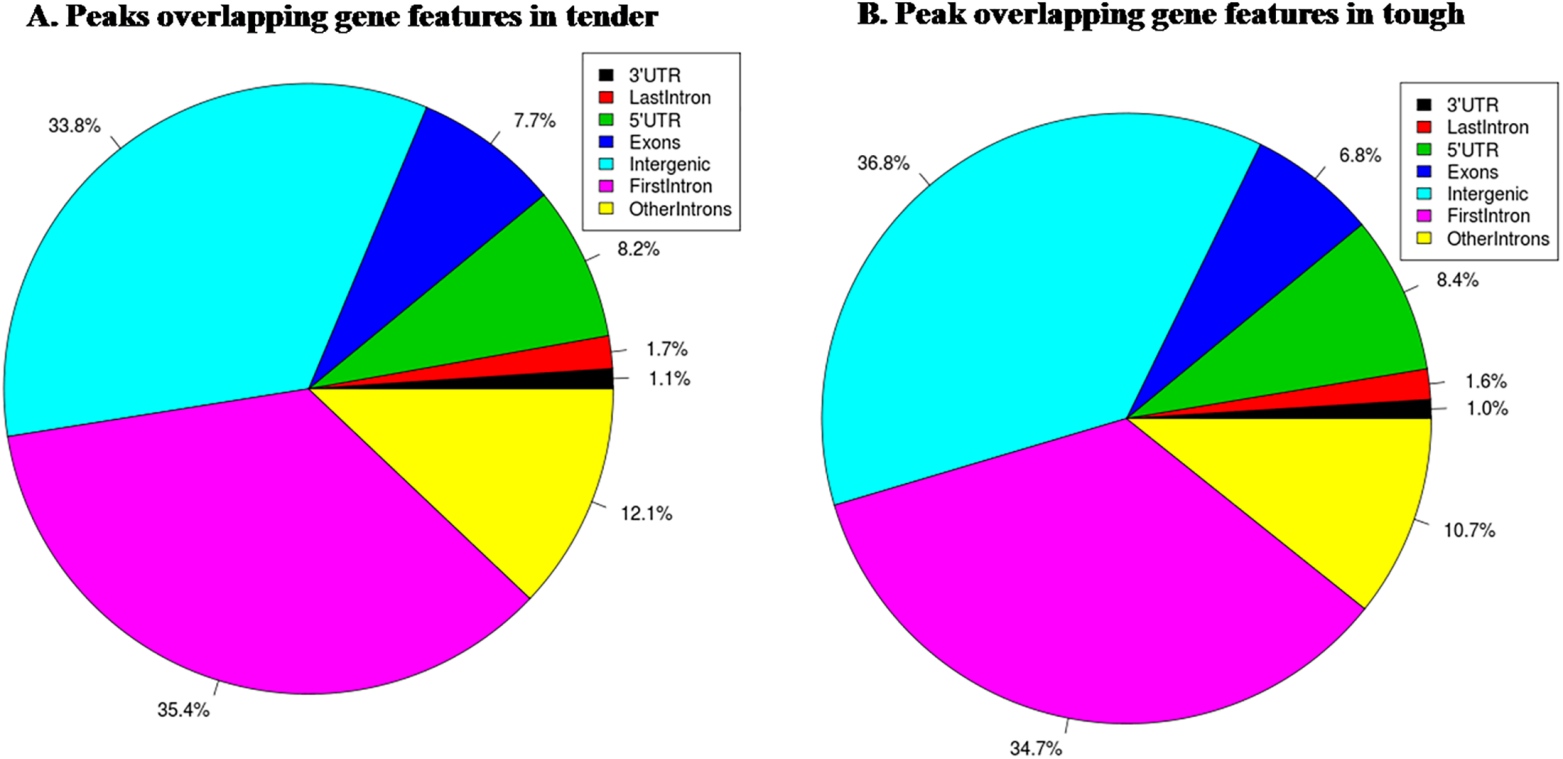
Relative H3K4me3 enrichment on functional parts of genes in LD. (A) Peaks overlapping gene feagures in tender group; (B) Peak overlapping gene features in tough group.

**Fig 4:**
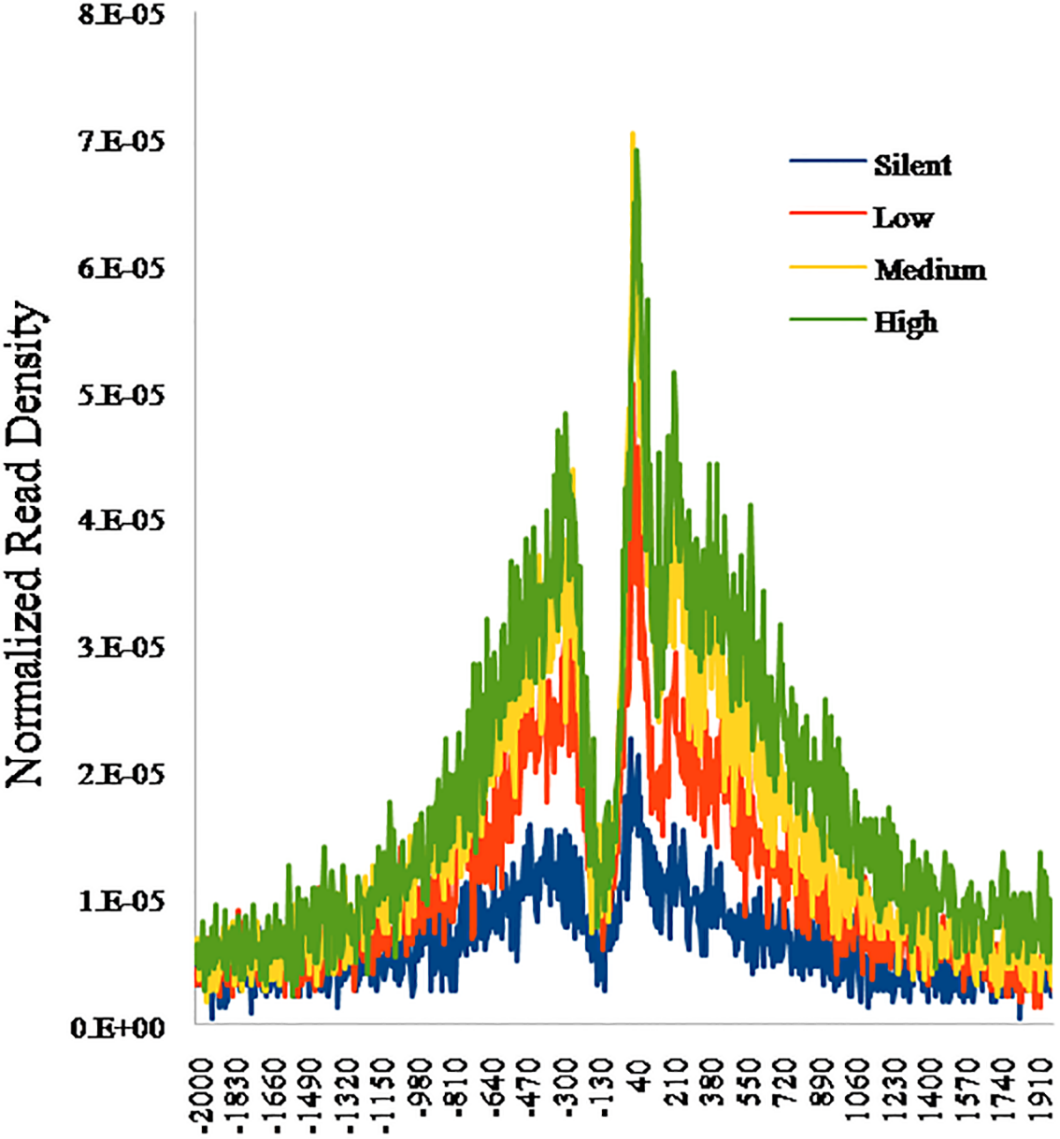
Histone modification enrichment in TSS (transcription start site) and gene body regions and its relationship with gene expression in 4 categories.

To explore the function of enriched regions, we searched the overlap between significant peaks with RefSeq and Ensembl genes. We identified 1,597 and 574 unique genes displaying H3K4me3 enrichment in the tender and tough group, respectively. Many of these genes possess pivotal functions in muscle differentiation and beef quality, implying that H3K4me3 plays regulatory roles in both events—muscle differentiation and beef quality variation—, as well. To identify whether H3K4me3 has preference for specific genomic elements, genes were subdivided into seven functional regions: 5’ UTR (Untranslated Regions), exons, first intron, last intron, other introns, 3’ UTR and intergenic regions (Fig 3). We found that distribution patterns of H3K4me3 on genes were almost identical between tender and tough groups with a majority of H3K4me3 markers occupying the first intron and intergenic regions. A survey of the distance between transcription start site (TSS) and the peak, calculated from its central point to the TSS of the nearest gene, showed that H3K4me3 locates mainly within ±1 kb around the TSS (Fig 4).

### The relationship of H3K4me3 and gene expression

To examine the relationship between H3K4me3 and gene activity in bovines, we evaluated gene expression levels of tender and tough groups using Agilent 4×44K bovine microarray [15]. Subsequently, we analyzed gene expression data along with ChIP-Seq data. To visualize H3K4me3 in gene promoters, we grouped all genes into four categories according to their expression levels (high, medium, low, and silent) and plotted the distribution of H3K4me3 levels around the TSS for each category (Fig 4). Results showed that H3K4me3 mainly enriches around the TSS and sharply declines with increasing distance from the TSS. Importantly, in these 4 categories, the genes with higher expression level also demonstrated larger amount of H3K4me3, indicating that H3K4me3 in promoter correlates highly and positively with gene expression.

**Fig 5:**
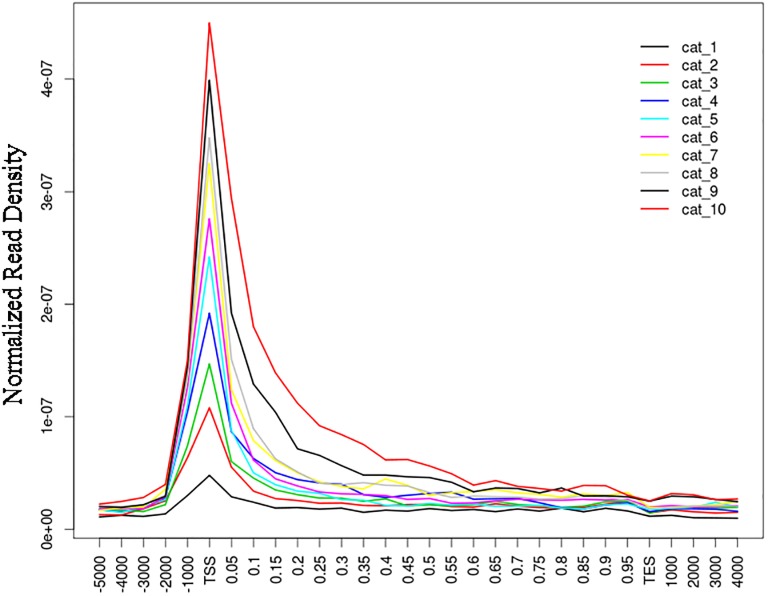
Histone modification enrichment in the entire gene structure and its relationship with gene expression in 10 categories.

To further test the relationship between gene expression and H3K4me3 on the entire gene structure, we grouped all the genes into ten categories regarding their expression level. These categories have arranged from the lowest category 1(cat1) to the highest category 10 (cat10). The H3K4me3 level for each group was plotted around TSS, promoter region (from -5000 to TSS), the gene body region, TTS (transcription termination site) and intergenic region (from 3000 to TTS). We scaled a percentage of length instead of the true length to solve the problem of plotting genes with different exon and intron length (Fig 5). The results were consistent with the histone marker profiles around the TSS region. Namely, the H3K4me3 mainly enriches around TSS and it occupancy at the whole-genome level positively correlates with gene expression.

### Clustering analysis

**Fig 6:**
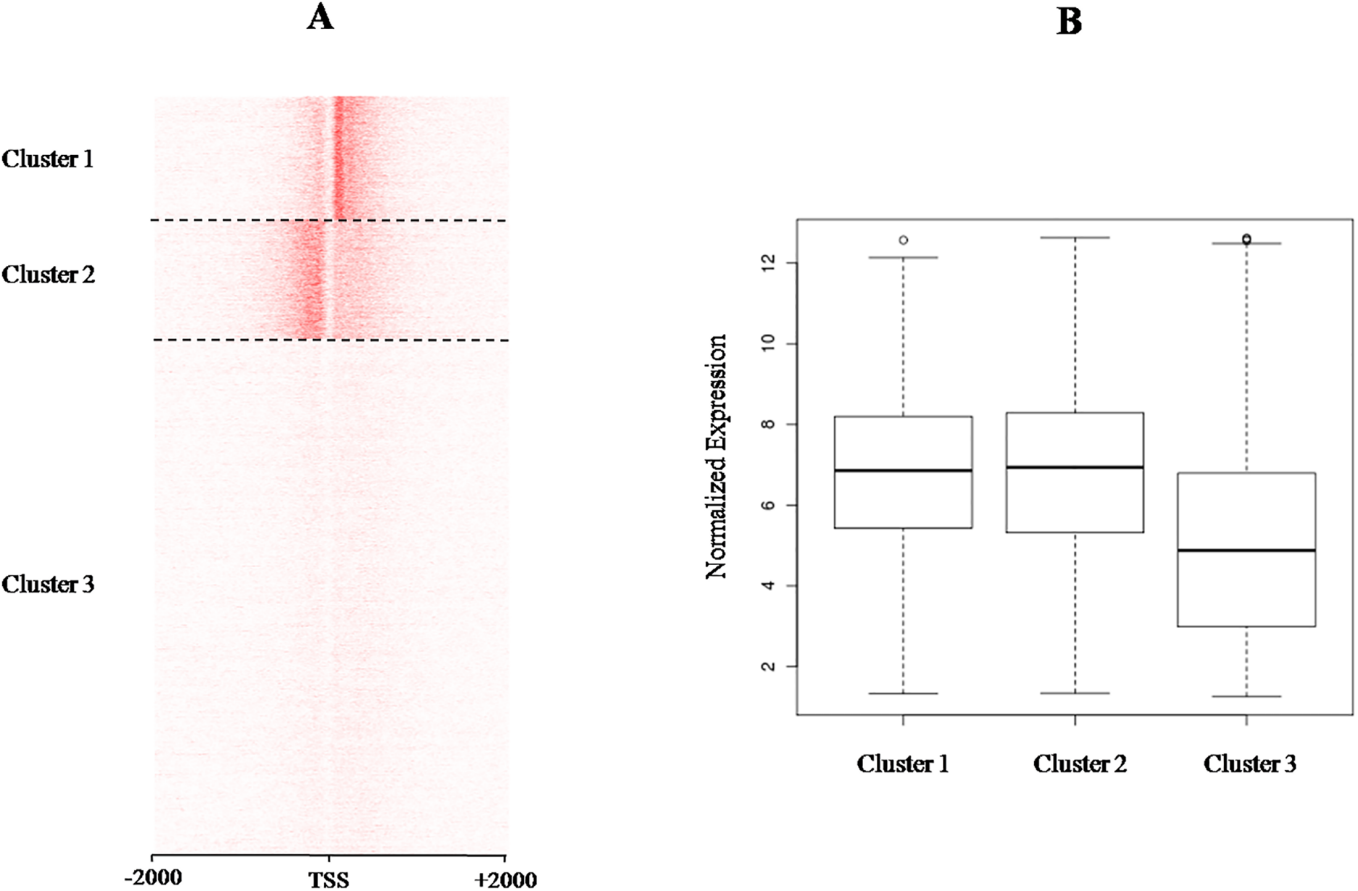
The relationship between H3K4me3 enrichment pattern and gene expression. (A) K-means clustering of different H3K4me3 enrichment pattern in promoters. (B) Box-plot for gene expression of different H3K4me3 enrichment pattern in LD.

In these LD samples, H3K4me3 mainly enriched in the promoter region of genes. Thus, we conducted k-means clustering for all the genes represented in the gene expression microarray to explore the H3K4me3 pattern in promoters. First we sorted all the genes by their expression value and plotted H3K4me3 level on promoters. The resulting profile vividly shows the positive association between gene expression and enrichment of H3K4me3 on the promoter (Fig 6A). Subsequently, we set k as 2, 3 and 4 to detect characteristic patterns within the histone modification profiles. The classification of 3 groups appeared to capture the major existing patterns (Fig 6A). The largest cluster (cluster 3) corresponded to genes without significant enrichment of H3K4me3, while the other two groups consisted of genes with striking enrichment of H3K4me3 on either side of the TSS. The corresponding expression of each gene class is shown in a box plot (Fig 6B) and revealed that the genes within cluster 3 exhibit lower expression levels than those in clusters 1 and 2, which, in turn, show similar overall expression. Thus, cluster 3 genes would likely maintain a silence stage in LD muscle in Angus cattle, while H3K4me3 plays regulatory roles in the expression of genes within clusters 1 and 2.

### Functional annotation of genes with H3K4me3 enriched promoters

It has been previously reported that H3K4me3 enriches the promoter region of active genes [16]. To investigate the function of this histone modification, we further explored the function of genes enriched with H3K4me3 in the promoter region, which was defined as the 4 kb region surrounding the TSS (-2000 to +2000 bp). In total, we found 1,597 and 574 genes in the corresponding tender and tough clusters. IPA analysis showed that genes in the tender group, involved in several pathways, such as connective tissue development and function, skeletal and muscular system development and function, tissue development, genetic disorder, skeletal and muscular disorders, connective tissue disorders, cellular growth and proliferation, cell morphology, cellular assembly and organization, cell signaling, lipid metabolism, molecular transport, carbohydrate metabolism, small molecule biochemistry, *etc* (S1 Table). And in the tough group, enriched genes associated with cell death, lipid metabolism, small molecule biochemistry, cellular compromise, drug metabolism, cellular development, hematopoiesis, genetic disorder, neurological disease, embryonic development, organ development, energy production, lipid metabolism, small molecule biochemistry, *etc* (S2 Table).

### Validation of ChIP, ChIP-Seq results by Q-PCR

It has reported that chaperonin containing TCP1, subunit 8 (CCT8)–an active gene—enriches with histone marker around TSS [17]. Additionally, in most cell types serpin peptidase inhibitor, clade A, member 1 (SERPINA1) associates with histone modifications correlated with silencing. Whereas it shows low levels of histone modifications, gene transcription activity increases. So we chose CCT8 and SERPINA1 as positive and negative control regions and performed q-PCR to check the ChIP quality before library construction. The ChIP q-PCR results showed that the H3K4me3 enrichment between CCT8 and SERPINA1 in tender and tough was 13.04-fold and 25.19-fold, respectively (S1 Fig). It means that ChIP quality was appropriate for further experiment. After sequencing and data analysis, we selected 3 regions with H3K4me3 peaks and performed ChIP Q-PCR to validate the ChIP-Seq results. The results showed good concordance between ChIP-Seq and ChIP-qPCR (S2 Fig).

## Discussion

Histone lysine methylation occurs on 6 lysine residues, lysine 4, 9, 27, 36 and 79 for histone H3, and lysine 20 for histone H4 [18]. Each of these can be mono-, di- or trimethylated and each methylation pattern can associate with gene activation or repression, depending on the site of methylation and the number of methyl groups added. For instance, H3K4me2 and H3K4me3 are associated with transcriptional activation, whereas H3K9me3 and H3K27me3 are related to transcriptional repression [19]. Histone methylation is catalyzed by several different enzyme families during myogenesis and muscle development. It has been reported that a histone methyltransferase, *SET and MYND domain containing 1* (smyD1), is required for myofibril organization and muscle contraction in zebrafish embryos and plays a critical role in myofibril development during myofiber maturation [20]. SETD3 constitutes a H3K4/K36 methyltransferase and participates in the transcriptional regulation of muscle cell differentiation [21]. Additionally, demethylation of H3K9me3 at the promoter of myogenin by an isoform of the histone demethylase, JMJD2A, probably induces muscle differentiation [22].

Using sequencing technology to analyze genome-wide chromatin changes has greatly improved our understanding of the relationship between epigenetics and gene expression. This global approach extends our view to a whole-genome-scale providing deep insights into histone modifications, in general. Here using genome-wide mRNA expression profiling and H3K4me3 enrichment profiling for tender and tough groups in LD of Angus, we obtained a global H3K4me3 map of the bovine genome and explored the impact of H3K4me3 on gene expression and beef tenderness. We found that the H3K4me3 markers on the whole genome or promoter regions displayed similar patterns in tender and tough groups, with high H3K4me3 enrichment surrounding the TSS and a significant decrease far from TSS. Moreover, our data confirmed a strong positive correlation between H3K4me3 levels and gene expression [23–25].

Tenderness is a very important determinant of beef quality, which is affected by many factors, including breed, nutritional status, muscle fiber type composition and postmortem aspects, such as temperature, sarcomere length and proteolysis. Of these factors, fiber types have been widely associated with meat tenderness, thus identifying the optimum fiber type composition remains as a hot spot in this field [26,27]. Muscle fiber type plays a fundamental role in modeling meat appearance, color stability, texture, taste, and tenderness as well [26,27]. Bovine skeletal muscle is mainly classified into two different categories, fast and slow, according to the differences in the ATPase properties of the myosin heavy chain (MHC) isoforms [12]. Slow muscle, otherwise known as oxidative muscle, associates with flavor or tenderness, while meat from fast fibers, which grow more rapidly, tends to be tougher and drier. Some research has focused on mechanisms to replace fast fibers with slow fibers to improve beef quality. It has been found that epigenetic factors, such as DNA methylation, histone acetylation and histone methylation, regulate skeletal muscle fiber types [19]. Researchers also found that H3 acetylation and H3K4me3 occupancy vary directly with the transcriptional activity of MHC genes in fast and slow fiber types [19]. Our results showed that myosin heavy chain 1(MYH1) enriched with H3K4me3 in tough group while H3K4me3 increased in myosin heavy chain 2 (MYH2) in tender group, implying that this histone marker may regulate myosin gene expression and the resulting variation of muscle fiber type composition, which is relevant for beef tenderness.

Myogenic Differentiation 1 (Myod1), a basic-helix-loop-helix (bHLH) transcription factor, constitutes a master regulatory gene of skeletal muscle differentiation and can induce skeletal muscle differentiation in cells from many different lineages [28]. Myogenic factor 5 (Myf5) and Myogenic factor 6 (Myf6) also express in skeletal muscle, and each has a crucial role in muscle specification and differentiation [28–32]. It has been found that the azacytidine-mediated demethylation of the myogenic differentiation (Myod) gene resulted in the conversion of 10T1/2 cells to skeletal muscle [33]. In our results, we found that Myod1, Myf5 and Myf6 were highly enriched with the H3K4me3 marker in tender and tough groups. However, each gene presents a different enrichment pattern, implying that this histone marker might involve in muscle differentiation and beef tenderness, partially by distinct marker arrangement of Myod1, Myf5 and Myf6.

Until all the factors involved in the beef tenderness variation are fully known and analyzed, our understanding of muscle differentiation regulated by the interplay of histone modification, transcription activity, and promoter function remains incomplete. Our current study indicates that knowledge about the general relationship of H3K4me3 and transcriptional activity is just the beginning of this exploration.

In summary, by using transcriptome and H3K4me3 enrichment profiling for tender and tough groups in LD of Angus, we first obtained a global bovine H3K4me3 map and explored the relationship of H3K4me3 on gene expression and beef quality. Although the H3K4me3 maps displayed similar patterns in tender and tough groups, with high H3K4me3 enrichment surrounding the TSS, we found that some genes were differently enriched with H3K4me3 markers in tender and tough groups implying that this histone marker may be intensively involved in muscle differentiation and beef quality. We need to further explore the biological mechanisms in the future.

## Materials and Methods

### Sample preparation and experimental design

Nineteen purebred Angus steers were obtained from the Wye Farm (Queenstown, MD). This herd has been closed for nearly 50 years, thus the steers shared similar genetic background and phenotypes. At approximately 12 months of age, the animals were serially harvested. Immediately after harvest, samples of longissimus dorsi (LD) from the right side of the carcass were obtained and placed in RNAlater solution (QIAGEN, Valencia, CA) at -80°C. The carcass was stored at 4°C for a total of 14 days. After this period, steaks were obtained from the LD at the level of the 12^th^ intercostal space and then frozen. For measurement of the WBSF, steaks were thawed at room temperature to an internal temperature of 4°C. Then, the steaks were cooked to a core temperature of 70°C using a George Foreman Lean Mean Fat Grilling Machine. The temperature of steaks was measured using an Oakton thermometer (Temp JKT Acorn series). The cooked steaks were then cooled down to room temperature. Using a sharp cylinder, especially designed for muscle, six cores (1.27 cm in diameter) were sampled parallel to the muscle fiber orientation. The Warner-Bratzler shear forces (WBSF) of the cores were obtained using an Instron #5442 Test Machine (Norwood, MA). The average WBSF of the six cores was calculated and used as the WBSF for the samples [34,35]. From these 19 steers, 4 with the lowest WBSF values (6.77±0.56 kg) were identified as tender and 5 samples with the largest WBSF values (19.93±0.39 kg) labeled as tough. Then both groups underwent further analysis. All procedures were approved by the University of Maryland Institutional Animal Care and Use Committee (Protocol # R-07-05).

### ChIP-Seq Library generation and sequencing

LD samples from 4 tender and 5 tough were chopped into pieces in cold 1×PBS, and then crosslinked and sonicated as described previously [17]. Briefly, after chromatin was prepared for all 9 samples, equal aliquots of chromatin from each individual were pooled to create tender or tough chromatins. Thirty microgram tender or tough chromatins were digested with Micrococcal Nuclease to produce mono-nucleosomes. Then chromatin was immunoprecipitated with H3K4me3 antibody (Millipore) and purified to obtain immunoprecipitated DNA. After checked the quality by PCR, the IP-DNA was end-repaired, added A and ligated with a pair of Solexa adaptors (Illumina, San Diego, CA). After performed PCR on the DNA using the adaptor primers, fragments with a length of approximately 200–400 bp (mononucleosome + adaptors) were isolated from the agarose gel. Sequencing analysis using the purified DNA was performed on the Solexa 1G Genome Analyzer (Illumina) following the manufacturer’s protocols. The data is available at NCBI Gene Expression Omnibus (GEO) (accession number: GSE61936).

### Microarray analysis

Gene expression was assessed in these samples with the 4×44K Bovine Gene Expression Microarray (Agilent, Santa Clara, CA) [36]. Total RNA was extracted from 4 tender and 5 tough samples using the Trizol RNA isolation method (Invitrogen, Carlsbad, CA) and purified with the QIAGEN RNeasy Mini Kit spin columns (QIAGEN, Valencia, CA). RNA was quantified on a spectrophotometer (ND-1000, Nanodrop, Wilmington, DE) and RNA integrity determined with a bioanalyzer (2100 Bioanalyzer, Agilent, Foster City, CA). The employed microarray was designed based on the whole bovine genome sequence, containing 21,475 unique probes, representing approximately 19,500 distinct bovine genes. Equal aliquots of RNA from all samples were pooled to create a common reference sample. Subsequently, two μg of RNA from each sample and the reference sample was reverse transcribed and labeled with Cy3 and Cy5 fluorochrome, respectively, using the Quick Amp Labeling Kit (Agilent). Then, 825 ng of the appropriate Cy3 and Cy5 labeled complementary RNAs (cRNA) were hybridized to the 4×44K microarray. According to the number of samples, a total of 9 arrays were hybridized. To combine the gene expression data with the sequencing data, we converted all Agilent probe IDs into RefSeq and Ensembl gene names. For genes with multiple probe sets in microarray data, the average expression of these probes was taken to represent the expression level of the gene.

### Mapping Read and summarizing count

Sequence reads obtained from the Solexa Genome Analyzer were aligned to the October 2007 version of the bovine genome (Baylor 4.0/bosTau4 using Maq version 0.7.1). Alignment policies of Maq were modified from default. A valid alignment could have a maximum of one mismatch and redundant reads were removed before further analysis to avoid amplification bias. If a read aligned equally well to multiple locations in the genome, this read was discarded to obtain the unique alignment. Only unique reads and unique aligned reads were chosen to perform further analysis. Read counts were summarized using non-overlapping windows of 200 bp for visualization on the UCSC genome browser. For the purpose of comparison between tender and tough group, summary counts in each sample were normalized to per million mapped reads in the corresponding sample.

### Peak identification

Summarized read counts were subjected to peak calling with SICER [37]. The source code was modified to support the bovine genome. Fragment length was specified to be about 190 bp as estimated from our ChIP-Seq experiments. A window size of 200 (default) and gap size of 400 bp was used for the analysis. The E-value for estimating significant peaks was set to 100.

### Overlap genes and peaks

Version 2007 of the RefSeq gene annotations were downloaded from UCSC genome browser. We defined a gene as being the region from 5000 bp upstream of 5’-end to 5000 bp downstream of 3’-end. To identify genes enriched with H3K4me3 marker, we aligned the peak with the genes. A gene was defined as enrichment if SICER identified peaks overlapped the gene promoter region.

RefSeq and Ensembl gene annotations were downloaded from UCSC Genome Browser and Ensembl Genome Browser. Because the RefSeq database only contained 13,974 genes, we included Ensembl genes in our analysis. The Ensembl database consists of 17,858 annotated genes, including validated and predicted genes. To maximize coverage of annotated genes we combined the two databases as follows: if a genomic region was annotated with both RefSeq and Ensembl genes, we used the RefSeq gene to annotate the region. As a result of this step, we obtained a non-redundant list of genes consisting of 13,974 RefSeq genes and 13,892 Ensembl genes.

### Genome-wide peak distribution and histone profiles

To calculate the genomic distribution of significant peaks we counted all peaks that overlapped with one of the following regions: promoter (TSS ± 1 kb), exons, introns, 5’ UTR and 3’ UTR. Genes were divided into ten categories based on their absolute expression. Histone modification profiles were calculated in 1 kb windows from 5 kb upstream to 5 kb downstream of the gene. For reads falling within the gene body, read counts were obtained in bins of 5% of the gene length. We also obtained read count profiles in 5 bp windows for 2 kb on either side of the TSS of the genes. The read counts were finally normalized to the total number of genes in the categories and the total number of reads in the sample.

### k-means clustering

K-means clustering used the region centered TSS from down-stream 2,000 bp to up-stream 2,000 bp. The signal was explored for all genes except for genes shorter than 2,000 bp. K means clustering was then conducted with Euclidean distance similarity metric. *k* (the number of the cluster) was set to 2, 3, 4 and 5. After the clusters were explored, the genes were sorted by their expression level and plotted in a heatmap. The log2 expression values from the cDNA microarrays were also plotted in a box plot to explore the expression patterns of different clusters.

### Function annotation of H3K4me3 enriched genes

Ingenuity Pathway Analysis (IPA) (Ingenuity System, www.ingenuity.com) was used to generate networks and assess statistically relevant biofunctions and canonical pathways. All the enriched genes was uploaded and mapped to corresponding expression genes in the Ingenuity knowledge database. The biofunctional analysis identified “molecular and cellular function” and “physiological system development and function”. Canonical Pathway Analysis identified pathways most significantly represented in the dataset. The significance between the dataset and the canonical pathway was measured using Fisher’s exact test obtaining a *p* value and a Benjamini-Hochberg correction for multiple testing was also applied.

### Validation of ChIP and ChIP-Seq results by Q-PCR

The quality of the ChIP and peaks found in ChIP-Seq result were validated by Q-PCR using an iCycler iQ PCR system (Bio-Rad). The Q PCR were performed in a final volume of 20 *μ*l with a QuantiTect SYBR Green PCR Kit (Qiagen) according to the manufacturer’s instructions. The primers for all of the genes analyzed are in S3 Table.

## Supporting Information

S1 Fig Validation of ChIP quality by Q-PCR. The quality of the ChIP was validated by quantitative PCRusing primers located on the promoter region of CCT8 and SERPINA1 respectively. The x-axis represents the samples while the y-axis represents the relative enrichment.
(TIF)

S2 Fig Validation of the three H3K4me3 peaks by Q-PCR. Primers were designed based on the predicted H3K4me3 peaks. The x-axis represents the samples while the y-axis represents the relative enrichment.
(TIF)

S1 Table Pathways that genes enriched with H3K4me3 involed in tender group. (XLS)

S2 Table Pathways that genes enriched with H3K4me3 involed in tough group. (XLS)

S3 Table Primers used in this experiment. (XLSX)

## References

[1] Warner R, Greenwood P, Pethick D, Ferguson D (2010) Genetic and environmental effects on meat quality. Meat Science 86: 171–183. 10.1016/j.meatsci.2010.04.042 20561754

[2] Robinson D, Ferguson D, Oddy V, Perry D, Thompson J (2001) Genetic and environmental influences on beef tenderness. Animal Production Science 41: 997–1003.

[3] Thompson J, Perry D, Daly B, Gardner G, Johnston D, et al (2006) Genetic and environmental effects on the muscle structure response post-mortem. Meat Science 74: 59–65. 10.1016/j.meatsci.2006.04.022 22062716

[4] Zhang CL, McKinsey TA, Olson EN (2002) Association of class II histone deacetylases with heterochromatin protein 1: potential role for histone methylation in control of muscle differentiation. Molecular and cellular biology 22: 7302–7312. 12242305 10.1128/MCB.22.20.7302-7312.2002 PMC139799

[5] Yang Q, Dahl M, Albertine K, Ramchandran R, Sun M, et al (2013) Role of histone deacetylases in regulation of phenotype of ovine newborn pulmonary arterial smooth muscle cells. Cell proliferation 46: 654–664. 10.1111/cpr.12076 24460719 PMC3904681

[6] McGee SL, Hargreaves M (2010) Histone modifications and skeletal muscle metabolic gene expression. Clinical and experimental pharmacology and physiology 37: 392–396. 10.1111/j.1440-1681.2009.05311.x 19793100

[7] Blais A, van Oevelen CJC, Margueron R, Acosta-Alvear D, Dynlacht BD (2007) Retinoblastoma tumor suppressor protein—dependent methylation of histone H3 lysine 27 is associated with irreversible cell cycle exit. The Journal of cell biology 179: 1399 10.1083/jcb.200705051 18166651 PMC2373492

[8] Caretti G, Di Padova M, Micales B, Lyons GE, Sartorelli V (2004) The Polycomb Ezh2 methyltransferase regulates muscle gene expression and skeletal muscle differentiation. Genes & development 18: 2627. 15520282 10.1101/gad.1241904 PMC525543

[9] Ait-Si-Ali S, Guasconi V, Fritsch L, Yahi H, Sekhri R, et al (2004) A Suv39h-dependent mechanism for silencing S-phase genes in differentiating but not in cycling cells. The EMBO Journal 23: 605–615. 14765126 10.1038/sj.emboj.7600074 PMC1271807

[10] Rampalli S, Li L, Mak E, Ge K, Brand M, et al (2007) p38 MAPK signaling regulates recruitment of Ash2L-containing methyltransferase complexes to specific genes during differentiation. Nat Struct Mol Biol 14: 1150–1156. 18026121 10.1038/nsmb1316 PMC4152845

[11] Perdiguero E, Sousa-Victor P, Ballestar E, Munoz-Canoves P (2009) Epigenetic regulation of myogenesis. Epigenetics 4: 541–550. 20009536 10.4161/epi.4.8.10258

[12] Pandorf CE, Haddad F, Wright C, Bodell PW, Baldwin KM (2009) Differential epigenetic modifications of histones at the myosin heavy chain genes in fast and slow skeletal muscle fibers and in response to muscle unloading. American Journal of Physiology-Cell Physiology 297: C6 10.1152/ajpcell.00075.2009 19369448 PMC2711647

[13] He Y, Yu Y, Zhang Y, Song J, Mitra A, et al (2012) Genome-wide bovine H3K27me3 modifications and the regulatory effects on genes expressions in peripheral blood lymphocytes. PLoS One 7: e39094 10.1371/journal.pone.0039094 22761725 PMC3386284

[14] Schones DE, Zhao K (2008) Genome-wide approaches to studying chromatin modifications. Nat Rev Genet 9: 179–191. 10.1038/nrg2270 18250624 PMC10882563

[15] Zhao C, Tian F, Yu Y, Luo J, Hu Q, et al (2012) Muscle transcriptomic analyses in Angus cattle with divergent tenderness. Mol Biol Rep 39: 4185–4193. 10.1007/s11033-011-1203-6 21901422

[16] Luo J, Mitra A, Tian F, Chang S, Zhang H, et al (2012) Histone methylation analysis and pathway predictions in chickens after MDV infection. PloS one 7: e41849 10.1371/journal.pone.0041849 22848633 PMC3406056

[17] Jin C, Zang C, Wei G, Cui K, Peng W, et al (2009) H3. 3/H2A. Z double variant—containing nucleosomes mark'nucleosome-free regions' of active promoters and other regulatory regions. Nature genetics 41: 941–945. 10.1038/ng.409 19633671 PMC3125718

[18] Greer EL, Shi Y (2012) Histone methylation: a dynamic mark in health, disease and inheritance. Nat Rev Genet 13: 343–357. 10.1038/nrg3173 22473383 PMC4073795

[19] Baar K Epigenetic control of skeletal muscle fibre type. Acta Physiologica 199: 477–487. 10.1111/j.1748-1716.2010.02121.x 20345412

[20] Tan X, Rotllant J, Li H, DeDeyne P, Du SJ (2006) SmyD1, a histone methyltransferase, is required for myofibril organization and muscle contraction in zebrafish embryos. Proceedings of the National Academy of Sciences of the United States of America 103: 2713 16477022 10.1073/pnas.0509503103 PMC1531647

[21] Eom GH, Kim KB, Kim JH, Kim JY, Kim JR, et al Histone methyltransferase SETD3 regulates muscle differentiation. Journal of Biological Chemistry. 10.1074/jbc.M110.203307 PMC3186363 21832073

[22] Verrier L, Escaffit F, Chailleux C, Trouche D, Vandromme M A New Isoform of the Histone Demethylase JMJD2A/KDM4A Is Required for Skeletal Muscle Differentiation. PLoS Genetics 7: e1001390 10.1371/journal.pgen.1001390 21694756 PMC3107188

[23] Barski A, Cuddapah S, Cui K, Roh TY, Schones DE, et al (2007) High-resolution profiling of histone methylations in the human genome. Cell 129: 823–837. 17512414 10.1016/j.cell.2007.05.009

[24] Ke XS, Qu Y, Rostad K, Li WC, Lin B, et al (2009) Genome-wide profiling of histone h3 lysine 4 and lysine 27 trimethylation reveals an epigenetic signature in prostate carcinogenesis. PLoS One 4: e4687 10.1371/journal.pone.0004687 19262738 PMC2650415

[25] Zhang X, Bernatavichute YV, Cokus S, Pellegrini M, Jacobsen SE (2009) Genome-wide analysis of mono-, di- and trimethylation of histone H3 lysine 4 in Arabidopsis thaliana. Genome Biol 10: R62 10.1186/gb-2009-10-6-r62 19508735 PMC2718496

[26] Klont RE, Brocks L, Eikelenboom G (1998) Muscle fibre type and meat quality. Meat Science 49: S219–S229. 22060713

[27] Maltin C, Balcerzak D, Tilley R, Delday M (2003) Determinants of meat quality: tenderness. Proceedings of the Nutrition Society 62: 337–348. 14506881 10.1079/pns2003248

[28] Molkentin JD, Olson EN (1996) Defining the regulatory networks for muscle development. Current Opinion in Genetics & Development 6: 445–453. 8791524 10.1016/s0959-437x(96)80066-9

[29] Puri PL, Sartorelli V (2000) Regulation of muscle regulatory factors by DNA binding, interacting proteins, and post transcriptional modifications. Journal of Cellular Physiology 185: 155–173. 11025438 10.1002/1097-4652(200011)185:2<155::AID-JCP1>3.0.CO;2-Z

[30] Perry RLS, Parker MH, Rudnicki MA (2001) Activated MEK1 binds the nuclear MyoD transcriptional complex to repress transactivation. Molecular cell 8: 291–301. 11545732 10.1016/s1097-2765(01)00302-1

[31] Buckingham M, Bajard L, Chang T, Daubas P, Hadchouel J, et al (2003) The formation of skeletal muscle: from somite to limb. Journal of anatomy 202: 59–68. 12587921 10.1046/j.1469-7580.2003.00139.x PMC1571050

[32] Pownall ME, Gustafsson MK, Emerson CP Jr (2002) Myogenic regulatory factors and the specification of muscle progenitors in vertebrate embryos. Annual review of cell and developmental biology 18: 747–783. 12142270 10.1146/annurev.cellbio.18.012502.105758

[33] Jones PA, Wolkowicz MJ, Rideout WM, Gonzales FA, Marziasz CM, et al (1990) De novo methylation of the MyoD1 CpG island during the establishment of immortal cell lines. Proceedings of the National Academy of Sciences 87: 6117–6121. 2385586 10.1073/pnas.87.16.6117 PMC54483

[34] Zapata I, Zerby HN, Wick M (2009) Functional proteomic analysis predicts beef tenderness and the tenderness differential. Journal of Agricultural and Food Chemistry 57: 4956–4963. 10.1021/jf900041j 19449808

[35] Sawdy JC, Kaiser SA, St-Pierre NR, Wick MP (2004) Myofibrillar 1-D fingerprints and myosin heavy chain MS analyses of beef loin at 36 h postmortem correlate with tenderness at 7 days. Meat Science 67: 421–426. 10.1016/j.meatsci.2003.11.014 22061516

[36] Zhao C, Tian F, Yu Y, Luo J, Hu Q, et al (2012) Muscle transcriptomic analyses in Angus cattle with divergent tenderness. Molecular Biology Reports 39: 4185–4193. 10.1007/s11033-011-1203-6 21901422

[37] Zang C, Schones DE, Zeng C, Cui K, Zhao K, et al (2009) A clustering approach for identification of enriched domains from histone modification ChIP-Seq data. Bioinformatics 25: 1952 10.1093/bioinformatics/btp340 19505939 PMC2732366

