# Supplementary figures and images for: Genome-Wide H3K4me3 Analysis in Angus Cattle with Divergent Tenderness
Source: PLoS One. 2015 Jun 18;10(6):e0115358. doi: 10.1371/journal.pone.0115358 (PMC4473007; doi:10.1371/journal.pone.0115358)

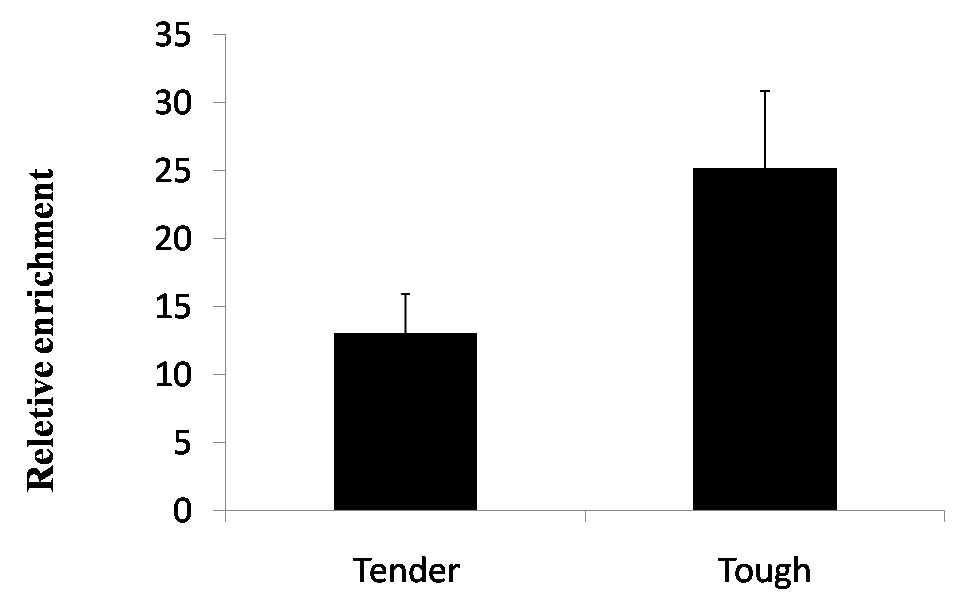

Supplement: S1 Fig — The quality of the ChIP was validated by quantitative PCRusing primers located on the promoter region of CCT8 and SERPINA1 respectively. The x-axis represents the samples while the y-axis represents the relative enrichment. (TIF) [file pone.0115358.s001.tif]

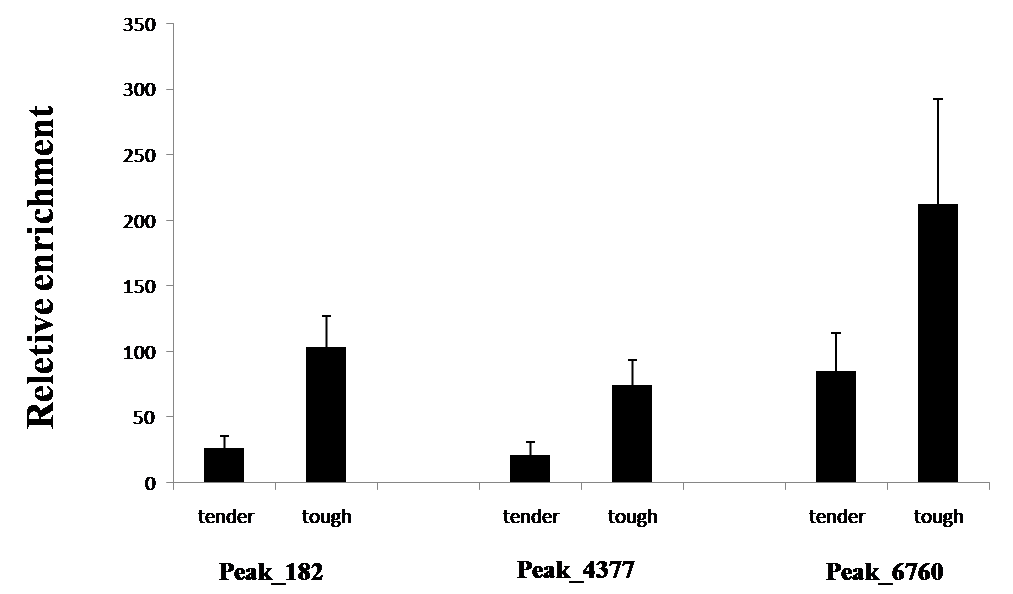

Supplement: S2 Fig — Primers were designed based on the predicted H3K4me3 peaks. The x-axis represents the samples while the y-axis represents the relative enrichment. (TIF) [file pone.0115358.s002.tif]
